# Facile Synthesis and Properties of Highly Porous Quartz Fiber-Reinforced Phenolic Resin Composites with High Strength

**DOI:** 10.3390/ma17112486

**Published:** 2024-05-21

**Authors:** Xin Tao, Yange Wan, Ruoyu Zhang, Yuqing Zhang, Yu Wang, Xiaolei Yu, Mingchao Wang

**Affiliations:** 1 College of Science, Civil Aviation University of China, 2898 Jinbei Road, Tianjin 300300, China; xtao@cauc.edu.cn (X.T.); mingchaowang0@163.com (M.W.); 2 School of Safety Science and Engineering, Civil Aviation University of China, Tianjin 300300, China; 3 Aviation Engineering Institute, Civil Aviation University of China, Tianjin 300300, China; 4 Dezhou Zhongke New Materials Co., Ltd., Dezhou 253011, China

**Keywords:** porous composite, quartz fiber felt, phenolic resin, microstructure, mechanical property

## Abstract

Lightweight and high-strength insulation materials have important application prospects in the aerospace, metallurgical, and nuclear industries. In this study, a highly porous silica fiber reinforced phenolic resin matrix composite was prepared by vacuum impregnation and atmospheric drying using quartz fiber needled felt as reinforcement and anhydrous ethanol as a pore-making agent. The effects of curing agent content on the structure, composition, density, and thermal conductivity of the composite were studied. The mechanical properties of the composite in the xy direction and z direction were analyzed. The results showed that this process can also produce porous phenolic resin (PR) with a density as low as 0.291 g/cm^3^, where spherical phenolic resin particles are interconnected to form a porous network structure with a particle size of about 5.43 μm. The fiber-reinforced porous PR had low density (0.372~0.397 g/cm^3^) and low thermal conductivity (0.085~0.095 W/m·K). The spherical phenolic resin particles inside the composite were well combined with the fiber at the interface and uniformly distributed in the fiber lap network. The composite possessed enhanced mechanical properties with compressive strength of 3.5–5.1 MPa in the xy direction and appeared as gradual compaction rather than destruction as the strain reached 30% in the z direction. This research provides a lightweight and high-strength insulation material with a simple preparation process and excellent performance.

## 1. Introduction

Porous polymers are widely used in construction as a thermal insulation material, which helps to keep warm air inside a building, save energy, and reduce construction noise transmission [1,2,3]. Out of concern for building safety, porous insulation materials with flame-retardant properties are highly desirable because they can prevent diffusion in the event of a fire and ensure adequate evacuation time [4,5,6]. Compared with polyurethane, polyvinyl chloride, or polystyrene foams, the porous phenolic resin has the advantages of being a strong flame retardant, exhibiting non-dripping performance during combustion, and having no toxic gases [7,8,9,10]. Seunghwan Wi et al. studied the fire resistance performance and toxicity characteristics of five types of insulation materials used in actual construction sites. Based on the total heat release and heat release rate, phenolic foam was the insulation material that satisfied the criteria of the non-combustible grade as a result of the flame-retardant performance evaluation. Moreover, porous phenolic resin has low thermal conductivity, stable structure, and acid/alkali corrosion resistance, which make it an excellent fire-retardant insulation material [11,12,13].

In addition to thermal insulation and fire-retarding functions, ideal insulation material for buildings also requires high strength and low brittleness to play a supporting and cushioning role, which can absorb part of the energy and weaken the impact of motion on the building, especially in natural disasters such as earthquakes. The molecular structure of ordinary phenolic resin is that two phenol molecules are connected by a methylene group, which reduces its chain rotation degree of freedom and increases steric hindrance. Hence, phenolic resin is brittle, and its wide application is thereby limited [14,15].

In recent decades, the toughening modification of phenolic resin has always been a hot topic of research [16,17,18,19]. It is an effective toughing method for adding fibers as reinforcement to prepare composite materials, which can improve the tear resistance, strength, toughness, and cut resistance, as well as reduce elongation at the break of the resin. Natural fibers are widely used because of their advantages of large yield, limited pollution, and renewable properties, and they include wood fiber [20], banana fiber [21], and cocoanut fiber [22], among others. However, natural plant fibers are polar, hydrophilic, and flammable, which increases the difficulty of preparing composite materials and limits its application in fireproof materials. Glass fiber is also a common reinforcement for its low cost, high-temperature resistance, and non-combustible properties in phenolic composites. Chopped short fiber [23], continuous long fiber [24], two-dimensional woven fiber fabric [25,26,27], and three-dimensional fiber braided body [28,29] are common forms of reinforcement. Among them, the use of three-dimensional reinforcement can effectively improve the wear resistance, shear strength, fracture toughness, and ablative resistance of the composite while avoiding the phenomenon of low interlayer bonding strength, and it can effectively inhibit the volume shrinkage during the curing process of phenolic resin [30,31,32]. Rui Yao et al. prepared a three-dimensional braided glass fiber-reinforced phenolic cryogel composite by microemulsion-templated sol–gel polymerization and freeze-drying methods, and the compressive and tensile strength had equally increased approximately 8 and 30 times after compounding with glass fiber [29]. Xinghong Zhang et al. prepared a completely lightweight 3D nanocomposites by vacuum impregnation and gel polymerization processing with a quartz fiber needled felt as the reinforcement and phenolic resin aerogel as the matrix, which showed enhanced toughness, low thermal conductivity, and fire-retardant properties [33,34]. With the increasing demand for such materials, more convenient and low-cost preparation processes are urgently needed and worth exploring in detail.

Here, a low-cost and simple preparation process of porous phenolic resin composites is introduced. The porous composites were prepared by in situ polymerization and atmospheric drying using silica fiber felt as three-dimensional reinforcement by impregnating a phenolic resin precursor solution. Compared with existing processes, neither complex drying methods such as freeze-drying nor time-consuming processes such as solvent replacement are required. Single-phase porous phenolic resin (PR) and quartz/porous PR composite with different curing agent content were prepared. The microstructure, basic physical properties, and chemical bonds of the samples were characterized. The thermal gravimetry, thermal conductivity, and mechanical properties of the samples were tested to explore the potential of porous phenolic resin as a fireproof insulation material for buildings.

## 2. Materials and Methods

### 2.1. Preparation of Porous Phenolic Resin Material

The first step involved the preparation of the precursor solution of phenolic resin. Resole phenolic resin (PR, 75%, Meisheng Engineering Plasticizing Co., Ltd., Yuyao, China) was dissolved in anhydrous alcohol (AR, Aladdin Reagent Co., Ltd., Shanghai, China) with a mass ratio of 30% and stirred magnetically for 60 min. The mixture was stirred continuously, and methenamine was added as a curing agent (Meisheng Engineering Plasticizing Co., Ltd., Yuyao, China) drop by drop so that the mass of methenamine was 10%, 15%, 20%, and 25% of PR. After stirring the precursor solution for another 60 min, the beaker was sealed and transferred to an airtight container and placed in an oven at 70 °C for 72 h to complete curing and aging. Finally, the aged samples were dried to constant weight in a blast oven at 50 °C and demolded to obtain porous phenolic resin (denoted as porous PR).

### 2.2. Preparation of Porous Fiber-Reinforced Phenolic Resin Material

**Figure 1:**
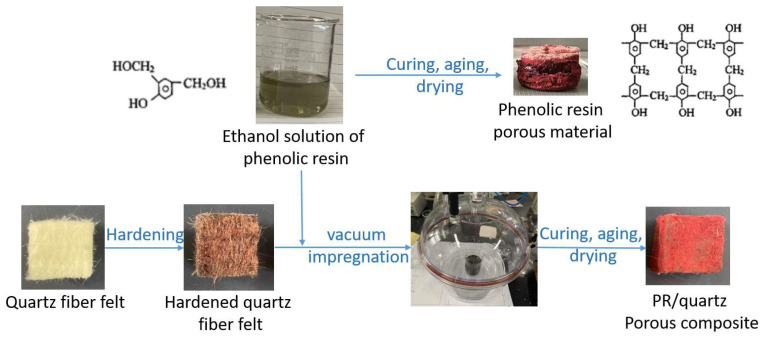
Preparation process diagram of quartz/porous PR composite.

The preparation process of porous fiber-reinforced phenolic resin is shown in Figure 1, and macroscopic photos of the samples during the process are also shown in the figure. Quartz fiber felt was purchased from Shenjiu Tianhang New Materials Co., Ltd. in Zhengzhou, China, and the density is 0.20 g/cm^3^. The felt was cut into cuboids with dimensions of 20 × 20 × 10 mm for use as the reinforced precast material. Firstly, the felt was impregnated in a precursor solution of phenolic resin with a concentration of 20% and then cured at 200 °C for 1 h to obtain the hardened quartz fiber felt. The density of the hardened quartz fiber felt is 0.25 g/cm^3^. Then, one drop of silane coupling agent (KH-550, Xingfei Long Chemical Co., Ltd., Jinan, China) was added to the precursor solution of phenolic resin, and the mixture was stirred continuously for 30 min. The concentration of the precursor solution was 30%, and the curing agent ratio was 15%, 17.5%, 20%, and 22.5%, respectively. Then, the hardened quartz fiber felt was impregnated in an 80 mL precursor solution, and the impregnation process kept the air pressure at 5 KPa for 30 min. Finally, the samples were subjected to curing, aging, and drying processes to obtain the quartz porous fiber-reinforced phenolic resin material (denoted as quartz/porous PR composite). The curing, aging, and drying processes were the same as those described in Section 2.1.

### 2.3. Characterization and Performance Testing

A scanning electron microscope (SEM, S-4800, Hitachi, Tokyo, Japan) was used for microstructural analysis. Thermogravimetric analysis was performed using a TGA instrument (STA-449C, Netzsch, Selb, Germany) with a heating speed of 20 °C/min in argon and air atmosphere, respectively. The molecular structure was identified by Fourier-transform infrared spectroscopy (FTIR, Nicolet iS5, Thermo Scientific, Waltham, MA, USA) in the wavenumber range of 4000 cm^−1^~400 cm^−1^. The samples were ground with dried potassium bromide (KBr) powder and pressed into a disc. The density of the samples was obtained by dividing the mass by volume. The volume shrinkage during the drying of the samples was calculated according to v% = 1 − *V*/*V*_0_ × 100%, where *V* is the volume of the dried sample, and *V*_0_ is the volume of the precursor solution. Ten samples were tested in each group to obtain the average value during the density and volume shrinkage test.

The thermal conductivity was tested using a Hot Disk TPS 2500 thermal constant analyzer at room temperature, for which two identical samples with dimensions of 30 mm × 30 mm × 10 mm were used, and the result was the average of the measurements at nine locations on the samples. An electronic universal mechanical testing machine (CSS-44001, Changchun Testing Machine Research Institute, Changchun, China) was used to measure the compressive strength of the samples with a displacement rate of 0.2 mm/min at room temperature. Since the quartz fiber needled felt had a two-dimensional planar random structure, in which the fibers were located on planes parallel to each other with random positions and orientations, the as-prepared quartz/porous PR composite would inherit the structural characteristics and show anisotropy of mechanical properties. Therefore, a compression test was conducted along the z direction (perpendicular to the plane) and the xy direction (parallel to the plane) separately. The compressive strength along the xy direction is the maximum value of stress at the elastic deformation stage, while the compressive strength along the z direction is the stress value when the strain is 10%. The Young’s modulus of the samples is the slope of the stress–strain curve of the elastic deformation stage during compression. To analyze mechanical properties, 10 samples were tested in each group.

## 3. Results

### 3.1. Structural and Physical Properties of Porous PR

**Figure 2:**
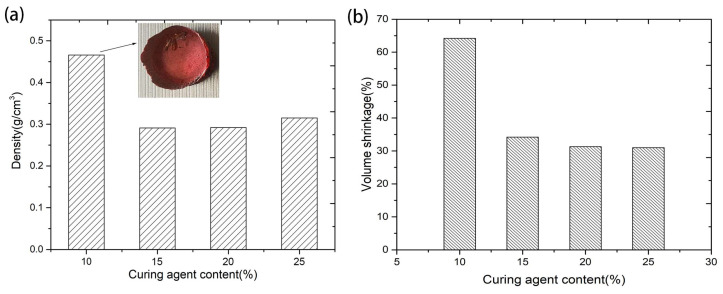
Density (**a**) and volume shrinkage during the drying process (**b**) of porous PR with different curing agent concentrations (inserted picture is a photo of the sample with 10% curing agent content).

After curing and aging, the fluidity of the precursor solution of phenolic resin was completely lost, and then the volumes of the PR blocks shrank to a certain extent after drying. The density and volume shrinkage during the drying of porous PR varied with the curing agent content, as shown in Figure 2a,b, respectively. The structure of the sample with 10% curing agent content collapsed significantly after drying, as shown in the inserted picture in Figure 2a. Therefore, the density was as high as 0.466 g/cm^3^, and the volume shrinkage was 64.2%, indicating that a curing agent content of 10% was obviously deficient. The samples with higher curing agent content showed less volume shrinkage after drying and had a very low density. The density values of porous PR with curing agent amounts of 15%, 20%, and 25% were 0.291 g/cm^3^, 0.292 g/cm^3^, and 0.315 g/cm^3^, respectively. The appropriate amount of the curing agent in the preparation of highly porous phenolic resin was significantly higher than that of dense phenolic resin. To a certain extent, increasing the amount of the curing agent can promote the curing of the resin, increase the strength of the aging network, and thus reduce the volume shrinkage during drying, but excessive curing agent will increase the density of porous PR.

**Figure 3:**
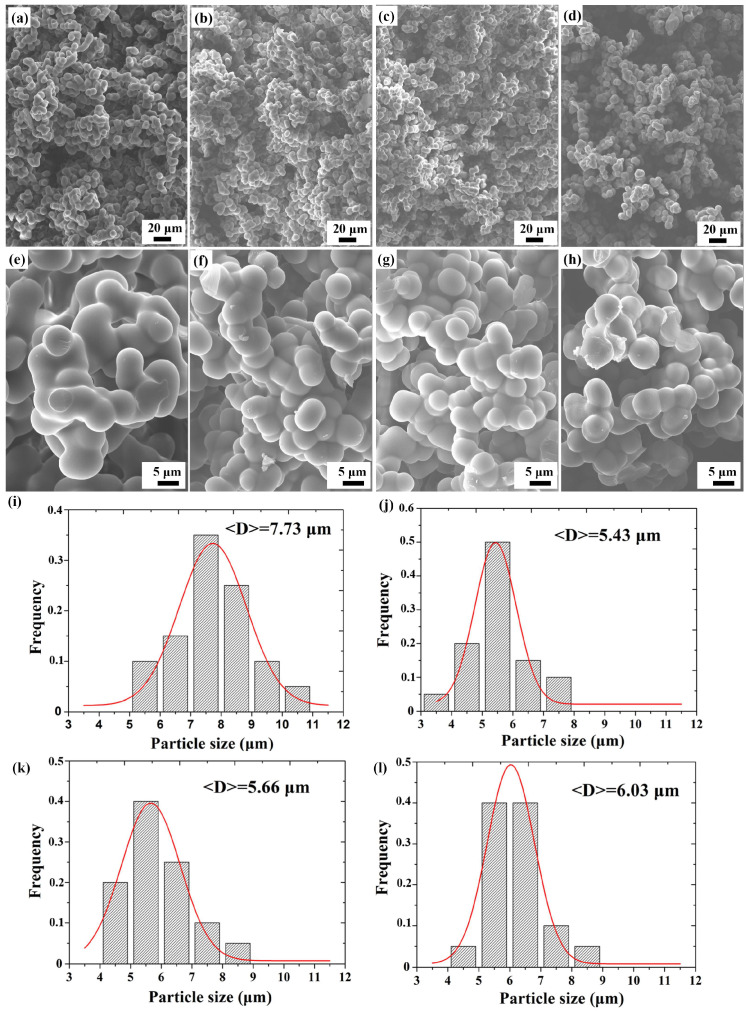
SEM images and particle size distribution curve of porous PR with curing agent content of 10% (**a**,**e**,**i**), 15% (**b**,**f**,**j**), 20% (**c**,**g**,**k**), and 25% (**d**,**h**,**l**).

The microstructure of porous PR with different curing agent contents is shown in Figure 3. In the interior of the porous PR, sphere particles are stacked into grape string polymers, which are connected to form a three-dimensional continuous mesh structure. A nano-measurer software program was used to randomly measure 50 particle sizes, shown in Figure 3e–h, and the statistic results and normal distribution fitting were obtained, which are shown in Figure 3i–l. The average particle sizes of porous PR with curing agent amounts of 10%, 15%, 20%, and 25% were 7.73 μm, 5.43 μm, 5.66 μm, and 6.03 μm, respectively. The porous PR structure with a curing agent content of 10% collapsed, indicating that the phase splitting of the resin and the pore-making agent (anhydrous ethanol) occurred in the curing and aging processes, and the high concentration of resin in the lower layer resulted in an increase in particle size. In other samples, the particle size increased slightly with the increase in the curing agent content, because the curing rate was accelerated with the increase in the amount of curing agent, which promoted particle growth.

### 3.2. Structural and Physical Properties of Quartz/Porous PR Composite

**Figure 4:**
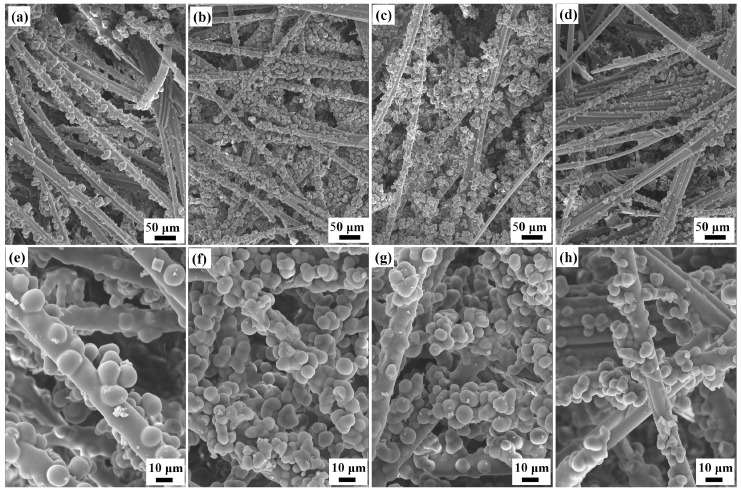
SEM images of the quartz/porous PR composite with curing agent concentrations of 15% (**a**,**e**), 17.5% (**b**,**f**), 20% (**c**,**g**), and 22.5% (**d**,**h**).

The microstructure of quartz/porous PR composite with different curing agent content is shown in Figure 4. Quartz fibers are generated, thus forming a porous network structure, and the phenolic resin particles are connected to each other at the neck and fill in the fiber network. The high-magnification SEM images (Figure 4e–h) show that the resin particles are closely attached to the fiber, and the interface is well bonded. Porous fibrous materials have a certain deformability under pressure, mainly through the bending deformation of fibers and the dislocation of fiber intersections. The phenolic resin particles wrap the surface of the fiber and gather at the fiber intersections, and therefore, the quartz/porous PR composite is expected to have increased strength and stiffness compared to quartz fiber. In the samples with curing agent amounts of 15% and 22.5%, more phenolic resin wraps around the fiber surface in the form of fiber coatings rather than accumulating particles. By contrast, in the samples with curing agent content of 17.5% and 20%, more phenolic resin exists in the form of particles on the surface of the fibers and in the gaps between the fibers.

**Figure 5:**
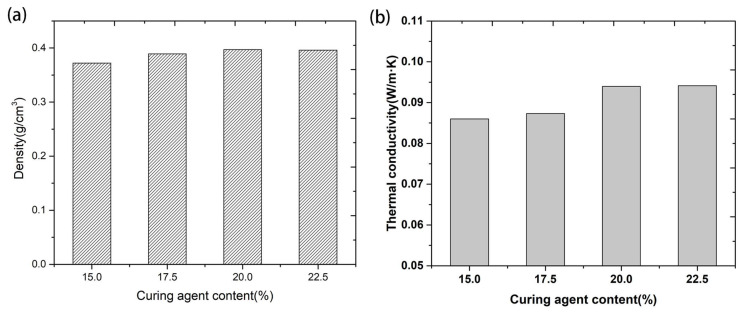
Density (**a**) and thermal conductivity (**b**) of the quartz/porous PR composite with different curing agent amounts.

The density of the quartz/porous PR composite with different curing agent content is shown in Figure 5a. The density increased slightly with the increase in the curing agent content and ranged from 0.372 to 0.398 g/cm^3^. Compared with porous PR, the quartz/porous PR composite showed no macroscopic volume shrinkage in the drying process. The fibrous network acted as a skeleton support, preventing the irregular aggregation of colloidal particles during drying and making the quartz/porous PR composite more symmetrical. The thermal conductivity of the quartz/porous PR composite is shown in Figure 5b. All the samples had rather low thermal conductivities, ranging from 0.086 to 0.094 W/mK, making the quartz/porous PR composite a potential candidate as a high-temperature thermal insulator. The low thermal conductivity is mainly attributed to the highly porous structure built by arbitrarily arranged quartz fibers and resin particles. With the increase in curing agent content from 15% to 22.5%, the slight increase in thermal conductivity is likely due to the increase in density.

### 3.3. Thermal and Composition Analyses of Quartz/Porous PR Composite

**Figure 6:**
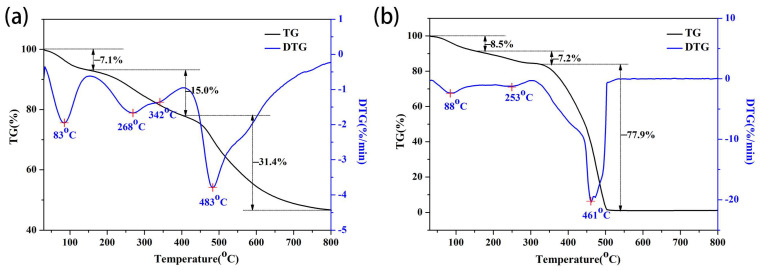
TG/DSC curves of the porous PR in argon (**a**) and air (**b**) atmospheres.

It is necessary to study the thermal stability of the composite as a potential insulating material. Since quartz fiber is stable below 1200 °C, only the thermal analysis of porous PR was conducted in an argon atmosphere and air atmosphere, as shown in Figure 6a,b, respectively. In the argon atmosphere, the total weight loss from room temperature to 1200 °C was 53.5%. The release of aliphatic amines, water, and formaldehyde contributed to the weight loss observed under 200 °C. The weight loss at 200–400 °C was due to the polycondensation reaction between functional groups, such as hydroxyl or hydroxymethyl. The most weight loss was observed during 400–800 °C, which is mainly related to the breaking of methylene ether and N-methylene bridges. Subsequently, hydrogen and oxygen atoms were removed from the aromatic structure, forming a polyaromatic structure and releasing hydrocarbons, carbon oxides, and hydrogen. The resin further carbonized at a relatively slow rate at temperatures above 800 °C. By contrast, in the air atmosphere, porous PR completely burned out at temperatures above 550 °C. The weight loss at low temperatures was similar to that in the argon environment, but the weight loss at 350–550 °C was significantly greater due to the presence of oxidation reactions.

**Figure 7:**
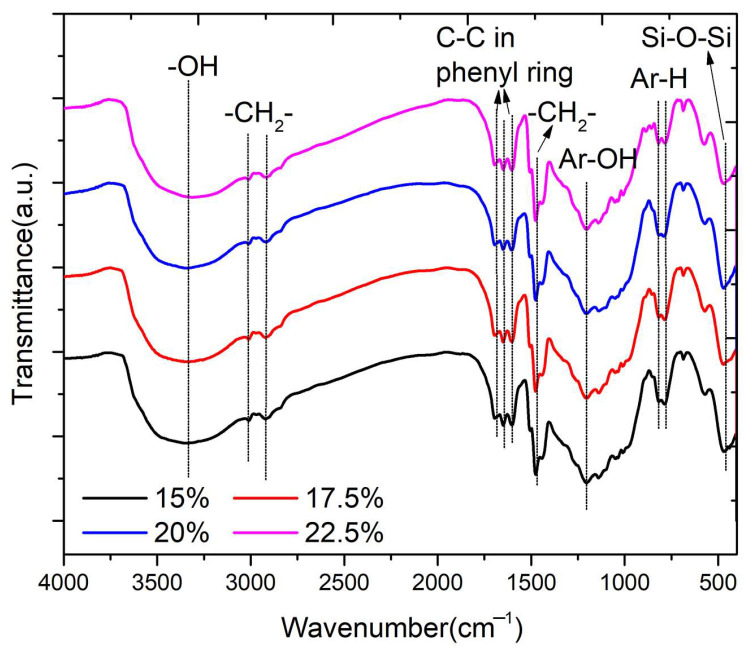
FTIR curves of the quartz/porous PR composite with different curing agent concentrations.

The FTIR curves of the quartz/porous PR composite with different curing agent amounts are shown in Figure 7. The presence of OH groups, which is attributed to the methylol and phenolic hydroxyl group, can be observed at band 3324–3358 cm^−1^. The C-H stretching vibration of methylene is in the range of 2810–2960 cm^−1^ [35,36]. The deformation vibrations of C-C bonds in phenolic groups absorb in the 1590–1690 cm^−1^ region. The bands related to aliphatic -CH_2_- asymmetric bending are observed in the range of 1480–1450 cm^−1^ [37,38]. The bands at 1204 cm^−1^ are related to the stretching vibration of phenolic C-O [39]. The deformation vibrations of the C-H bond in benzene rings give absorption bands in the 770–740 cm^−1^ range. These absorption peaks are the characteristics of fully cured phenolic resins. The asymmetric bending vibration peak of Si-O-Si is observed at 459 cm^−1^, which is attributed to the SiO_2_ in quartz fiber. The position and intensity of absorption peaks of the samples with different curing agent amounts were almost the same, suggesting that the curing degree of phenolic resin after constant temperature curing at 70 °C is independent of the amount of curing agent at the range of 15–22.5%.

### 3.4. Mechanical Performance of Quartz/Porous PR Composite

**Figure 8:**
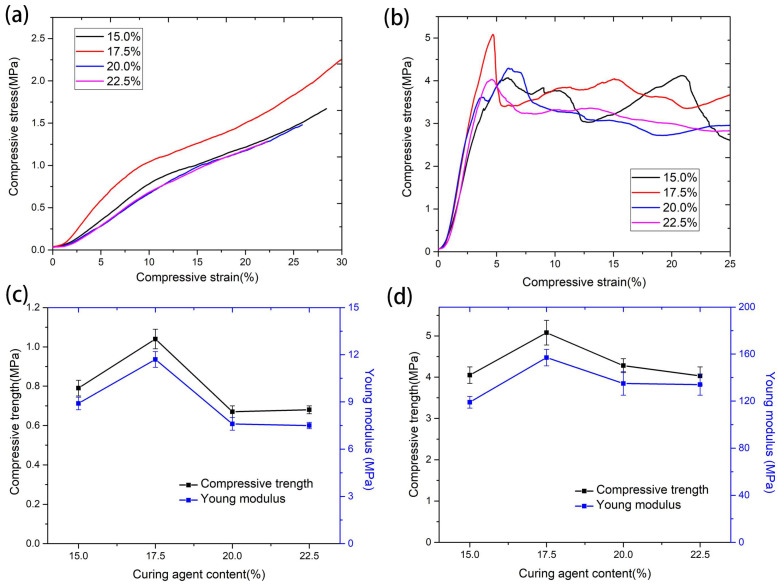
Compressive stress–strain curves of the quartz/porous PR composite along the z direction (**a**) and the xy direction (**b**). Compressive strength and Young’s modulus varied with the curing agent content along the z direction (**c**) and the xy direction (**d**).

Figure 8a shows the representative compressive stress–strain curves for the quartz/porous PR composites that were compressed along the z direction. The initial sections of the curves increase linearly, and the elastic deformation of this stage is believed to occur due to the elastic bending and rotation of fibers [40]. In the subsequent section, the stress increased at a lower speed with increasing strain. Since most quartz fibers were perpendicular to the compressive force, they were inclined to be tightly compressed and thus absorb more external energy. It should be noted that the stress did not decrease, and the samples were not damaged as the strain reached 50% in the z direction. Figure 8b shows the compressive stress–strain curves of the samples along the xy direction. The linear growth sections of the curves were still associated with the elastic deformation of the samples. After the stress reached its maximum, the stress decreased with strain, indicating that plastic deformation occurred because of the fracture and debonding of the fibers, as well as the structural collapse of interconnected resin particles.

The compressive stress and Young’s modulus of the samples with different curing agent content along the z direction and xy direction are shown in Figure 8c,d, respectively. One can see that the compressive strength (4.03–5.08 MPa) and Young’s modulus (119–157 MPa) of the samples along the xy direction are higher than those along the z direction (0.67–1.04 MPa and 7.5–11.7 MPa). The mechanical properties of fiber-reinforced composites are largely related to the distribution and orientation of fibers. Since most of the fibers were distributed within the xy plane and formed a wide variety of angles relative to the compressive force, it is helpful to enhance the compressive strength and Young’s modulus through fiber buckling.

In addition, the compressive strength and Young’s modulus of the quartz/porous PR composite increased first and then decreased with the increase in curing agent content, in both the xy direction and z direction. Additionally, the samples with a curing agent content of 17.5% exhibited optimal mechanical properties. For pure phenolic resin, the strength and Young’s modulus mainly depend on the chemical bond force of the main chain and the secondary valence bond between molecules. With the increase in curing agent content, the crosslinking density of phenolic resin increased, and moderate crosslinking formed a network that could effectively strengthen the connection between molecular chains, so the strength and Young’s modulus increased. However, the increase in crosslinking density also led to a reduction in the free volume of the molecular chains; thus, their movement between the crosslinking points was limited, and the deformation ability of the material was inhibited, leading to decreased strength. Moreover, the mechanical properties of fiber-reinforced resin composites are greatly affected by the properties of the interface, which affects the load transfer between the fibers and the matrix [41]. The content of the curing agent affects the behavior of phenolic resin in the composite. When the resin was mainly attached to the surface of the fiber, thus forming a continuous fiber coating (as shown in Figure 4e,h), the bearing capacity of the resin phase diminished, and the deformability of the fibers was reduced, so the mechanical properties of the composite were relatively poor [42].

Table 1 compares the comprehensive properties of the quartz/porous PR composite in this study with various fire-proof insulation materials based on phenolic resin, including phenolic foams, inorganic modified and fiber-reinforced phenolic aerogels, and various forms of fiber-reinforced phenolic resin composites. Compared with phenolic foams, the quartz/porous PR composite has lower thermal conductivity and significantly higher strength and fracture toughness. At the same time, the temperature resistance of the composite is improved due to the addition of temperature-resistant quartz fiber, and the thermal weight loss and the ablation regression rate decrease. Compared with phenolic aerogels, although the quartz/porous PR composite has higher density and thermal conductivity, its strength is dozens of times higher than ordinary phenolic aerogels and higher than fiber-reinforced phenolic aerogels; moreover, it has obvious advantages in terms of the complexity of the preparation process, preparation cost, and production time. Dense fiber-reinforced phenolic composites are also commonly used fire-proof materials and can be used for load-bearing conditions due to their superior mechanical properties. However, their density and thermal conductivity are much higher than that of quartz/porous PR composites, and they are not suitable for occasions where high insulation performance is required. In general, the as-prepared quartz/porous PR composite simultaneously has excellent thermal insulation and mechanical properties, a feature that is not available in other systems.

In addition, this work presents a low-cost and simple preparation process for phenolic resin composites. We compared the quartz/porous PR composite in this study with other phenolic resin-based fire-proof insulation composites from the aspects of raw material cost, manufacturing equipment cost, and equipment operation cost. As reinforcement, glass fiber felt has a lower price than glass fiber fabric and various carbon fiber-reinforced materials. There is no need for complex and expensive drying facilities, such as supercritical drying and freeze-drying equipment, which are required when preparing aerogel composites. Moreover, the preparation process is completed at normal pressure below 100 °C, which reduces energy consumption caused by heating and pressurization in most phenolic composites.

## 4. Conclusions

In this study, highly porous fiber-reinforced phenolic resin composites were prepared by vacuum impregnation, in situ polymerization, and atmospheric drying using quartz fiber needled felt as reinforcement and anhydrous ethanol as the pore-making agent. A dual continuous network structure was formed in the as-prepared composite, where spherical phenolic resin particles with a particle size of several microns were interconnected, thus forming a porous organic network, and the two-dimensional planar random structured quartz fibers overlapped each other, leading to the formation of a porous inorganic network. The as-prepared composite possessed low density (0.372~0.397 g/cm^3^), low thermal conductivity (0.085~0.095 W/m·K), high compressive strength (3.5~5.1 MPa) in the xy direction, and superior deformable ability in the z direction. The curing agent content needed in the porous phenolic composite was higher than that in phenolic resin, and the composite had the best performance when the curing agent content was 17.5%. This method has the advantages of low cost and simple process, and the finished product can be used as insulation and fire-retarding material for buildings with certain load-bearing and shock absorption requirements due to its superior and comprehensive thermal insulation and mechanical properties.

## Figures and Tables

**Table 1:** Comprehensive properties of various fire-proof insulation materials based on phenolic resin.

Product Classification	Density (g/cm^3^)	Strength (MPa)	Thermal Conductivity (W/m·K)	Preparation Method	Reference
Phenolic foams	0.04–0.06	0.09–0.26 (compressive strength)	0.1–0.16	Blow foaming	[43]
Phenolic–silica aerogels	0.02–0.06	<0.025 (compressive strength)	0.025–0.045	Co-polymerization based on chitosan-templated method	[11]
Fiber-reinforced phenolic aerogel	0.31–0.36	0.43–2.84 (compressive strength)	0.017–0.031	Vacuum impregnation and gel polymerization processing	[33]
Glass fabric/phenolic composite	1.5–2.0	150–250 (tensile strength)	-	Hand lay-up method, hot-compression molding	[1,5]
Natural fiber/phenolic composites	1.4–2.0	0.3–1.2 (internal bonding strength)	-	Mixing blending and hot-compression molding	[20,44]
Quartz/porous PR composite in this work	0.37–0.40	0.67–5.1 (compressive strength)	0.085–0.095	Vacuum impregnation and gel polymerization processing	-

## Data Availability

Data are contained within the article.
